# The impact of gamification on smoking cessation: A systematic review and meta-analysis

**DOI:** 10.18332/tid/203937

**Published:** 2025-06-26

**Authors:** Dina Syazana Ho Imran Ho, Fathiah Jabir, Siti Norain Sallahuddin, Nur Atiqah Mohd Ahwan, Ghaneshinee Sathiyaseelan, Mohamad Izzi Zahari, Mohd Rohaizat Hassan, Azmawati Mohammed Nawi

**Affiliations:** 1 Department of Public Health Medicine, Faculty of Medicine, National University of Malaysia, Kuala Lumpur, Malaysia; 2 Department of Public Health Medicine, Universiti Kebangsaan Malaysia, Kuala Lumpur, Malaysia; 3 University of Cyberjaya, Cyberjaya, Malaysia

**Keywords:** smoking cessation, gamification, systematic review, meta-analysis, mHealth

## Abstract

**INTRODUCTION:**

The rise of mobile health (mHealth) has led to increased adoption of mobile apps for smoking cessation. The integration of gamification has been found to be positively associated with higher app engagement, smokers’ self-efficacy and motivation to quit. This systematic review and meta-analysis aimed to identify and assess the game elements incorporated into smoking cessation applications and evaluate the effectiveness of gamified interventions on smoking cessation outcomes.

**METHODS:**

A comprehensive search was conducted in PubMed, Web of Science, Scopus, Cochrane Central Register of Controlled Trials (CENTRAL), and WHO International Clinical Trials Registry Platform (ICRTP) and ClinicalTrials.gov databases from inception to November 2024. Eligible studies included interventional trials comparing gamification-based smoking cessation strategies with non-gamified control groups. Two independent reviewers performed study screening, data extraction, and quality assessment using the Cochrane Handbook for Systematic Reviews. Outcomes were narratively synthesized, and comparable studies were pooled based on follow-up time and abstinence duration. A random-effects meta-analysis assessed smoking abstinence using relative risk (RR) as the effect measure. Heterogeneity was evaluated using Cochran’s Q and I^2^ index. The study was registered with the International Prospective Register of Systematic Reviews (PROSPERO) under the reference number CRD42024611631.

**RESULTS:**

Fifteen randomized controlled trials (RCTs) with a total of 5075 participants met the inclusion criteria. Key gamification elements included competition, milestone recognition, storytelling, and rewards. The pooled meta-analysis demonstrated a significant impact of gamified interventions on smoking abstinence. The strongest effects were observed within the first six months of intervention (RR=1.91; 95% CI: 1.47–2.47, p<0.001). Long-term effects remained significant beyond six months (RR=1.37; 95% CI: 1.05–1.79, p=0.02). Sensitivity analysis confirmed the robustness of these findings.

**CONCLUSIONS:**

Gamification-based interventions significantly improve smoking cessation outcomes, particularly in the short-term. However, the diminishing effects over time highlight the importance of long-term engagement. While these findings are promising, limitations such as heterogeneity in follow-up periods, reliance on some self-reported outcomes, and the inability to isolate specific gamification components may affect the generalizability of results. Leveraging gamification’s potential can still transform smoking cessation efforts, offering scalable and engaging solutions for lasting behavioral change.

## INTRODUCTION

Tobacco use remains a significant global health challenge, accounting for 8.7 million deaths annually and leading to tens of millions of preventable illnesses, making it the leading cause of preventable death worldwide^1^. Over the past three decades, smoking has caused more than 200 million deaths and imposed economic costs exceeding US$1 trillion each year^2^. While smoking prevalence has declined significantly among both males (27.5%) and females (37.7%) since 1990, population growth has resulted in an increase in the absolute number of smokers, with 1.14 billion individuals consuming 7.41 trillion cigarette-equivalents globally in 2019^2^. Smoking remains the leading risk factor for death among males, contributing to 20.2% of male mortality and 200 million disability-adjusted life years (DALYs) globally^2^. Recognizing the central role of tobacco in non-communicable diseases (NCDs), tobacco control has been identified as pivotal to achieving the WHO’s global NCD targets, including a 25% reduction in premature mortality by 2025 and a one-third reduction by 2030 under the Sustainable Development Goals^1^. Without intensified interventions, the annual burden of smoking-related deaths and DALYs is projected to rise in the coming decades.

Quitting smoking remains a significant challenge, with research suggesting that over 30 attempts may be required for success^3^. Traditional behavioral strategies for smoking cessation, such as counselling, financial incentives, and tailored support, have shown varied effectiveness^4^. In recent years, digital health technologies, particularly mobile health (mHealth) and electronic health (eHealth) solutions, have emerged as accessible and scalable options for providing behavioral support to individuals attempting to quit smoking^5-7^. The rapid growth of smartphone ownership, with over 8 billion mobile subscriptions globally as of 2018, has amplified the reach of these interventions, particularly in low-resource settings where access to in-person services is limited^3,4^. Despite their potential, mHealth interventions such as text messaging and app-based programs often face significant challenges, including low user engagement and inconsistent adherence. These shortcomings are frequently attributed to a lack of personalization and interactive features, which are critical to sustaining user interest and motivation and underscore the need for more innovative approaches to enhance the effectiveness of these interventions^4-6^. Herein lies the potential for gamification.

Gamification is the application of game elements in non-game contexts and has emerged as a promising strategy to enhance engagement and motivation in behavioral interventions^8^. Examples of game elements include achievement badges, goal setting, progress tracking, levels, and social sharing, all of which have demonstrated the ability to positively influence cognitive components of behavioral change^9^. Grounded in behavioral change theories such as self-determination theory and goal-setting theory, gamification provides users with intrinsic motivation and incremental milestones, which can improve confidence and task performance^8,9^. Preliminary evidence suggests that gamification may effectively address common challenges in mHealth smoking cessation interventions, such as low engagement and retention. However, most existing research has relied on qualitative approaches, limiting the ability to quantify the effectiveness of gamification on smoking cessation^8,10^. Additionally, understanding the composition of game elements within gamified smoking cessation applications is largely unexplored. Therefore, there is a critical need to synthesize existing evidence on the impact of gamification on smoking cessation outcomes and to identify the game elements driving behavioral change.

This systematic review and meta-analysis aims to: 1) Identify and assess the game elements incorporated into smoking cessation applications, and 2) Evaluate the effectiveness of gamified interventions on smoking cessation outcomes. By addressing these gaps, this review seeks to provide actionable insights for the design and implementation of gamified mHealth tools that can support smokers in achieving long-term cessation.

## METHODS

This systematic review and meta-analysis were conducted in accordance with the 2020 Preferred Reporting Items for Systematic Reviews and Meta-analyses (PRISMA) guidelines^11^. The study was registered with the International Prospective Register of Systematic Reviews (PROSPERO) under the reference number CRD42024611631.

### Data sources and search strategies

A systematic literature search for relevant publications from inception up until November 2024 was done from the following databases: PubMed, Scopus, and Web of Science. A comprehensive search was also conducted of the following trial databases: Cochrane Central Register of Controlled Trials (CENTRAL), WHO International Clinical Trials Registry Platform (ICRTP), and ClinicalTrials.gov. Combinations of keywords and synonyms representing population (smokers), intervention (gamification), and outcomes (smoking cessation and abstinence) were used as parts of the search strategy. The search strings used for this review are given in Supplementary file Table 1.

### Eligibility criteria

Studies were eligible for inclusion if they were non-randomized and randomized clinical trials (RCTs) and if they met other criteria based on the participants, interventions, comparators, and outcomes framework and assessed smoking abstinence. Both full-scale RCTs and pilot RCTs were included. No limitations were imposed regarding language or year of study implementation. In addition, only peer-reviewed original articles were included, while reviews or abstracts from conference proceedings were excluded. This review was guided by the following PICO question: ‘Among individuals who smoke (Population), do gamification-based interventions (Intervention), compared to non-gamified or standard smoking cessation interventions (Comparison), lead to improved smoking abstinence (Outcome)?’.

### Participants

This review includes participants of any age who were current smokers during their enrolment in the study. For this study, a ‘smoker’ was defined as a tobacco product user, in accordance with the definition provided by the Society for Research on Nicotine and Tobacco (SRNT) Treatment Research Network^12^. This includes all combustible tobacco products (cigarettes, cigars, little cigars), other tobacco products (heated-not-burn products) and alternative products [e-cigarettes and electronic nicotine delivery system (ENDS)].

### Intervention

Studies with interventions that incorporated one or more gamification elements for smoking cessation and abstinence were included. Gamification was defined as ‘the use of game design elements in non-game contexts’^13^. This encompassed the use of game-based mechanics (desired interactions over repeated uses, time, or between users of various components and other game-based elements to encourage progress and achievement) and game-based design elements (parts of a game that make it interesting, engaging, and compelling to players) in non-game settings to engage users and encourage achievement of desired outcomes through the motivation of users.

### Comparators

Only studies with a control group, either no intervention or non-gamified interventions, were included in this review. Studies that incorporated gaming elements for their control group were excluded.

### Outcomes

Studies reporting smoking abstinence measured at any period of follow-up were included. Other outcomes, such as user engagement or satisfaction rate, were not recorded.

### Screening

The screening process involved two independent reviewers who assessed each study’s title and abstract against the previously outlined eligibility criteria. Subsequently, the full texts of potentially relevant studies were thoroughly evaluated to determine their suitability for inclusion. In instances of disagreement, consultation with a third reviewer was sought.

### Data extraction

Data extraction was done independently by two reviewers, and any disagreement was resolved by a third reviewer. Data extraction was done using a pre-determined template on Google Sheets for easy access. Extracted data included study characteristics (year of publication, authors, country, study design, sampled population: age, and sample size), intervention (duration and description of intervention, follow-up period, and game elements), control characteristics, and outcome measurements.

### Risk of bias assessment

The risk of bias for each included study was assessed by two reviewers using the revised Cochrane Risk of Bias Tool for Randomized Trials (RoB 2) via five prespecified domains^14^: 1) bias arising from the randomization process, 2) bias due to deviations from intended interventions, 3) bias due to missing outcome data, 4) bias in the measurement of the outcome, and 5) bias in the selection of the reported result^14^. Disagreements were resolved between the two reviewers in the presence of a third reviewer.

### Data synthesis and statistical analysis

A qualitative data synthesis was carried out to describe and summarize the game elements incorporated in each intervention. For quantitative analysis, a meta-analysis was done using Review Manager 5.4. Risk ratios (RRs) were used to express the effect sizes for dichotomous data and their 95% confidence intervals (CIs) were calculated for analysis. For consistency, crude relative risks (RRs) were used as the effect measure across all included studies. When studies reported odds ratios (ORs) without providing RRs, we extracted the raw event data (number of participants achieving abstinence in each group) and calculated unadjusted RRs to enable uniform pooling of results. Random-effects meta-analysis were performed using DerSimonian estimator. All statistical tests were two-tailed, and a p<0.05 was considered indicative of statistical significance. Heterogeneity between studies were evaluated by performing a standard χ^2^ test with a significance level of 0.05. To assess heterogeneity, the I^2^ statistic was also calculated, and categorized as: low, <25%; moderate, 25–75%; and high, >75%. Forest plots were used to present the pooled estimates of risk ratios and the corresponding 95% confidence intervals. In addition, publication bias was assessed by observing the symmetry of funnel plots.

## RESULTS

**Figure 1:**
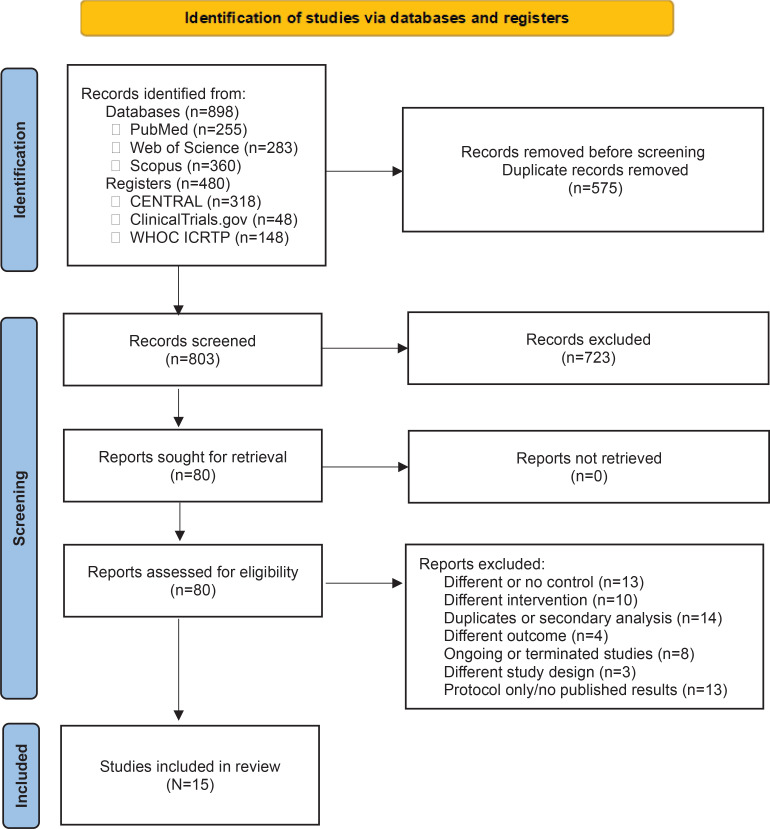
PRISMA 2020 flow diagram for new systematic reviews which included searches of databases and registers only

A total of n=898 records were identified through the initial database search. After removing duplicates, 80 full-text articles were assessed for eligibility, as shown in Figure 1. Following a detailed review of the full-texts, 65 studies were excluded for various reasons, including duplicate or secondary analyses (n=14), studies with different or no control group (n=13), protocol-only publications with no available results (n=13), studies with different interventions (n=10), ongoing or terminated studies (n=8), studies measuring different outcomes (n=4), and ineligible study designs (n=3). Ultimately, 15 studies met the inclusion criteria and were included in this systematic review and meta-analysis^15-29^.

The included studies were assessed for methodological quality using the RoB2 tool, as illustrated in Supplementary file Figures 1-4. Most studies were individually randomized, parallel-group randomized controlled trials, while one study was classified as a cluster-randomized trial^23^. In terms of risk of bias, two studies were categorized as high risk^17,19^. Nine studies were considered to have a moderate risk of bias^15-18,21,23,24,26,28^. The remaining five studies were classified as having a low risk of bias^20,22,25,27,29^.

### Characteristics of included studies

Table 1 summarizes the characteristics of the 15 studies included in this review, detailing the year of publication, country of study, study design, participant characteristics, intervention and control groups, study duration, and primary cessation-related outcomes. The majority of studies were published after 2020, with a smaller proportion conducted in earlier years. Most studies originated from high-income countries, with the United States being the most represented, followed by Spain, Australia, the Netherlands, and the United Kingdom. All studies included in this review employed a randomized controlled trial (RCT) design.

**Table 1:** Characteristics of included randomized controlled trials (RCTs) evaluating gamification-based smoking cessation interventions

*Authors* *Year* *Country*	*Total* *n*	Participants	*Age (years)* *mean (SD)*	Smoker definition	Study period	Intervention group	Control group	Primary cessation-related outcome
Hicks et al.^15^ 2017 USA	11 IG 5 CG 6	Adult smokers with current post-traumatic stress disorder (PTSD)	IG 53.2 (10.5) CG 54.3 (9.5)	Smoked more than 10 cigarettes per day for one year or more	6 months	‘QUIT4EVER’ program with Stay Quit Coach app. The program combined mobile platform for contingency management, counselling, medication and Stay Quit Coach app. The Stay Quit Coach app support smoking abstinence through personalized plan.	QUIT4EVER program without Stay Quit Coach app	Prolonged abstinence 1-2 weeks, salivary cotinine verified
Marin-Gomez et al.^16^ 2019 Spain	42 IG 21 CG 21	Pregnant women	IG 31.67 (4.9) CG 30.43 (6.02)	Smoked more than one cigarette per day	9 months	‘Tobbstop’ mobile app to support smoking cessation through gamification, e-health strategies, and mobile learning. Using the game app, the players are to clean and purify a metaphorical polluted island which symbolize a smoker’s body, as they go through the process of detoxification. Integrated with standard counselling, Tobbstop offers an engaging, holistic approach to quitting smoking.	Standard smoking cessation care counselling	Continuous abstinence until delivery, CO verified
Krebs et al.^17^ 2019 USA	38 IG 20 CG 18	Cancer patients scheduled for surgical treatment	Overall 57.11 (9.6)	Smoked cigarettes within the past 30 days	1 month	Combined ‘QuitIT’ game (Smoking Cues Coping Skills Game) and Standard care. The game designed as a narrative game with 10 episodes featuring various characters and smoking-related triggers, such as social events or stress.	Standard care which consists of four telephone or bedside counselling sessions and in-house print cessation educational materials.	7-day abstinence at 1 month follow-up, salivary cotinine verified
Peiris et al.^18^ 2019 Australia	49 IG 25 CG 24	Aboriginal or Torres Strait Islander aged more than 16 years	IG 42 (14) CG 42 (14)	Self-proclaimed smoker	6 months	A mobile app comprising a personalized profile and quit plan, text and in-app motivational messages, and a challenge feature allowing users to ‘compete’ with others. All smoking cessation support services available to them.	All smoking cessation support services available to them except for the app.	Continuous abstinence at 6 months follow-up, CO verified
Scholten et al.^19^ 2019 Netherlands	144 IG 72 CG 72	Youth smoker	IG 19.15 (2.25) CG 19.63 (2.59)	At least a weekly smoker	3 months	‘HitnRun’ mobile game which is a runner-style smoking cessation intervention that incorporated short, engaging gameplay to distract from cravings, personalized prompts for motivation, and team-based peer interaction for support and accountability. Team-based interactions were done via Google Hangout.	Self-help brochure (What you should know about quitting smoking) by the Trimbos Institute.	Self-reported 24-hour abstinence at 4 weeks and 3 months follow-up*
Vilardaga et al.^20^ 2019 USA	62 IG 33 CG 29	Adult smokers with serious mental illness	IG 46.1 (11.3) CG 45.6 (10.9)	Smoked five or more cigarettes per day with a carbon monoxide (CO) breath test reading of more than 6 parts per million	16 weeks	The ‘Learn to Quit’ app features 28 modules focused on ACT-based smoking cessation, incorporating USCPG, psychoeducation, and tips for nicotine replacement therapy. The app features two types of modules: lesson modules for teaching cessation content and skills modules for practice and it incorporates gamification elements to enhance retention and engagement.	QuitGuide app which is a smartphone application developed by the NCI and delivers USCPG contents for smoking cessation. It has 4 sections, namely ‘Thinking about quitting’, ‘Preparing to quit’, ‘Quitting’, and ‘Staying quit’.	30-day abstinence at 16 weeks follow-up, CO verified
Chen et al.^21^ 2020 China	80 IG 40 CG 40	Adult Chinese male smokers	IG 32.4 (6.0) CG 31.4 (5.1)	Smoked any type of tobacco products on a daily basis or occasionally	6 weeks	Full version of ‘SCAMPI’ program (Chinese-language smoking cessation program) that includes quitting plans, calculator to record quitting benefits, progress calendar, gamification to facilitate quitting, information on smoking harm, motivational messages, standardized test for levels of nicotine dependence and lung health, and social platform for social support.	Restricted version of SCAMPI program (Static WeChat page of contacts for standard smoking cessation care)	30-day abstinence at 6 weeks follow-up, salivary cotinine verified
Bricker et al.^22^ 2020 USA	2415 IG 1214 CG 1201	Adult smokers	IG 38.2 (10.8) CG 38.3 (11.0)	Smoked 5 or more cigarettes per day or concurrently using any other tobacco products (e.g. e-cigarettes) for the past year	12 months	‘iCanQuit’ app which is a self-paced interactive smartphone application that teaches acceptance and commitment therapy (ACT) skills for coping with smoking urges, staying motivate, and preventing relapse.	QuitGuide app which is a smartphone application developed by the NCI and delivers USCPG contents for smoking cessation. It has 4 sections, namely ‘Thinking about quitting’, ‘Preparing to quit’, ‘Quitting’, and ‘Staying quit’.	Self-reported 30-days abstinence at 12 months follow-up
Pallejà-Millán et al.^23^ 2020 Spain	773 IG 284 CG 318	Adult smoker	IG 42.2 (10.2) CG 48.8 (11.0)	Smoked at least 10 cigarettes per day	12 months	‘Tobbstop’ mobile app to support smoking cessation through gamification, e-health strategies, and mobile learning. Using the game app, the players are to clean and purify a metaphorical polluted island which symbolize a smoker’s body, as they go through the process of detoxification. Integrated with standard counselling, Tobbstop offers an engaging, holistic approach to quitting smoking.	Recommendations and information from health professionals based on standard guidelines of clinical practice.	Self-reported continuous abstinence at 3 and 12 months follow-up
Peek et al.^24^ 2021 Australia	64 IG 31 CG 33	Adult smokers	IG 61 (9) CG 62 (8)	Ever smoker	3 months	My QuitBuddy is an app personalized to help people quit smoking, using educational and motivational tools that motivate user across 4 functional domains: rational health, emotional, social and gamification. It also provides direct links to Quitline. Standard-of-care smoking cessation interventions provided by their primary care provider.	Smoking cessation webpage hosted by Queensland Department of Health (Quit HQ). Registration website for 12-week program of support emails containing health advice, motivational stories, and Quitline links. Standard-of-care smoking cessation interventions provided by their primary care provider.	Self-reported 12-weeks abstinence at 3 months follow-up
Houston et al.^25^ 2022 USA	433 IG 213 CG 220	Adult smokers	Overall 54 (13)	Actively smoking cigarettes	6 months	‘Take A Break (TAB)’ intervention which includes motivational messaging, challenge quizzes, brief abstinence goal setting, mobile health apps for craving management and rewards; combined with NRT.	NRT only	7-day abstinence at 6 months follow-up, CO verified
Schnall et al.^26^ 2022 USA	40 IG 20 CG 20	Adult smoker with HIV	IG 53.4 (10.2) CG 54.0 (8.5)	Smoked 5 or more cigarettes per day for the past 30 days	12 weeks	‘Lumme Quit Smoking’ app paired with a smartwatch that is able to detect smoking motion. The app was able to predict cravings, target users with notification to prevent individuals from smoking, refine the notifications for each user, and display their change in smoking behavior and money saved in a smoking diary. Users were also able to see their quit plan with their assigned quit date for 2 weeks after baseline, along with smoking trends, supportive tips, and badges earned from the amount of money saved. Also received smoking cessation counselling sessions and NRT.	Standard smoking cessation counselling session and NRT.	7-day abstinence at 12 weeks follow-up, CO verified
Marler et al.^27^ 2022 USA	188 IG 94 CG 94	Resident of the United States	IG 46.6 (9.2) CG 46.1 (8.2)	Current daily cigarette smoker (≥5 cigarettes per day) for the past 12 months	26 weeks	‘Pivot’ app which include interactive educational activities, the ability to log cigarettes, set a quit date, create a quit plan, complete practice quits (1-24 hours in duration), play educational games, watch educational videos, interact with one’s dedicated human coach via in-app text messaging, view CO breath sample values and trends, learn about and then order NRT, access the moderated web-based Pivot community discussion forum, share goals and progress with the web-based Pivot community discussion forum or one’s social network via SMS text messaging or email, and complete daily check-ins after quit date.	QuitGuide app which is a smartphone application developed by the NCI and delivers USCPG contents for smoking cessation. It has 4 sections, namely ‘Thinking about quitting’, ‘Preparing to quit’, ‘Quitting’, and ‘Staying quit’.	Continuous abstinence at 26 weeks follow-up, CO verified
Webb et al.^28^ 2022 UK	530 IG 265 CG 265	Adult smoker	IG 40 (12) CG 42 (12)	Smoked >5 cigarettes a day for the past year	52 weeks	‘Quit Genius’ app which is a 52-week digital clinician-assisted CBT intervention. The app is comprised with self-guided CBT content, coupled with a quit coach who provided asynchronous messaging to reinforce CBT skills and promoting smoking cessation including encouraging medication adherence, goal setting and self-monitoring. The app collected user data that tailored the pace and content to each participant.	Very Brief Advice (VBA) which is a simple form of advice designed to increase referrals to smoking cessation services. VBA follows the structure of ‘Ask’ patients about their tobacco use, ‘Advise’ them that the best method of quitting is with a combination of medication and behavioral support, and ‘Act’ by supporting them with making a quit attempt using available cessation support.	Self-reported 7-day abstinence at 4 weeks follow-up
Chen et al.^29^ 2024 China	206 IG 101 CG 105	Adult smokers who own a smartphone and have experience in using apps	IG 34.62 (8.03) CG 34.30 (7.04)	Smoked more than 100 cigarettes in their lifetime and currently smokes 5 or more cigarettes a day	4 weeks	‘Cognitive behavioral therapy (CBT)-based’ app designed to empower users to quit smoking through a comprehensive and personalized approach. The app offers tools for users to create a tailored quit plan, track cigarette consumption, cravings and money saved, while providing insights into health achievements such as blood circulation and oxygen levels. The app also has an in-built support system to connect users with friends and family, and an emergency SOS feature to help them remain smoke-free.	Regular SMS text messages to thank them for their participation and to remind them to complete their smoking status at each point.	Self-reported continuous abstinence at 4 weeks follow-up

* In the event that the primary outcome was not related to our research objective, the closest relevant outcome was selected. NR: not reported. IG: intervention group. CG: control group. CBT: Cognitive Behavioral Therapy. App: application. SD: standard deviation. NCI: National Cancer Institute. USCPG: US Clinical Practice Guidelines.

Our review included a total of 5075 participants across 15 studies. Participant characteristics varied across the studies. While most studies focused on adult smokers aged ≥18 years, two studies included younger participants, with Peiris et al. (2019)^18^ and Scholten et al. (2019)^19^ examining individuals aged ≥16 years. Among the adult populations, the mean age of participants ranged from late 30s to early 50s, with some studies focusing on younger adults in their late teens and others on older populations in their 60s.

The definition of smoking status varied across studies, though most classified smokers based on the number of cigarettes consumed per day. A common threshold was ≥5 cigarettes per day, with other studies including broader definitions such as self-reported smoking within a specified timeframe. The study durations also differed, with commonly used follow-up periods of three^15,19,22,24,26,27^, six^22,27,28^, and twelve months^22,23,28^.

The interventions evaluated in the included studies were gamification-based smoking cessation applications, integrating behavioral strategies with interactive and engaging digital tools. One study^17^ combined a gamification application with standard care. In the control groups, most studies provided standard smoking cessation support, such as counselling, educational materials, or brief interventions, while three studies used the QuitGuide application as a comparator^20,22,27^.

Primary cessation-related outcomes were primarily assessed through continuous abstinence measures or point prevalence abstinence at specified time points.

Continuous abstinence was reported in several studies^16,18,23,24,27-29^, while others assessed tobacco cessation using point prevalence abstinence at seven days^15^ or 30 days^20,22,27^. In terms of continuous abstinence, the shortest duration was assessed at four weeks^18,29^, while the longest duration was one year^23,28^. Biochemical verification, such as carbon monoxide breath testing^15,16,18,20,23,25-28^ or salivary cotinine measurements^15,17,21^, was utilized in some studies to confirm self-reported abstinence.

### Gamification elements and theoretical frameworks

Table 2 summarizes the gamification elements integrated into the smoking cessation interventions examined in this review, highlighting the primary strategies used to enhance engagement and adherence. The most common gamification elements included competition^18,19-21,25^, gameplay with rewards^16,17,20,23,25,26,28^, milestone recognition^26,28,29^, and storytelling^20,29^, each serving different motivational functions. Several interventions incorporated a gameplay and reward system, where participants earned points, badges, or in-game progress for achieving smoking cessation milestones. Others utilized competition-based mechanics, encouraging users to engage with peers or leaderboards to maintain motivation^18,19,21,25^. Milestone recognition was also a key feature, with interventions rewarding users for achieving abstinence goals over time. A smaller number of studies employed storytelling-based gamification, where smoking cessation was framed within a narrative to enhance engagement and relatability^20^.

**Table 2:** Gamification elements and theoretical frameworks in smoking cessation interventions

*Authors* *Year* *Country*	Intervention	Theory (if applicable)	Gamification Element
Hicks et al.^15^ 2017 USA	QUIT4EVER program with Stay Quit Coach app	Cognitive Behavioral Therapy	Personalized Interactive Tools Tailored plans to help users manage smoking urges Interactive tools with progress tracking and educational content
Marin-Gomez et al.^16^ 2019 Spain	Learn to Quit	Acceptance and Commitment Therapy	Storytelling and Skill Development Storytelling to make the journey relatable Game challenges to encourage skill practice Token-based rewards for task completion
Krebs et al.^17^ 2019 USA	Social mobile game HitnRun	Transactional Analysis Theory	Team Dynamics and Competition Runner-style game with point collection Cooperative and competitive team-based play Rewards tied to team participation
Peiris et al.^18^ 2019 Australia	Smoking Cues Coping Skills Game (QuitIT)	Social Cognitive Theory	Interactive Scenario Play and Rewards Interactive scenarios for managing smoking urges Monitored via a ‘urge to smoke’ meter Points, badges, and real-life coping cards as rewards
Scholten et al.^19^ 2019 Netherlands	A mobile app with personalized profile, quit plan, and competition feature	Not stated	Challenges and Competition Weekly challenges to foster motivation Competitive feature to challenge and engage with other users
Vilardaga et al.^20^ 2019 USA	Tobbstop app	Not stated	Metaphorical Gameplay and Rewards Detoxify a polluted island as a metaphor for body detoxification Visual progress through island enhancements Rewards to sustain engagement
Chen et al.^21^ 2020 China	iCanQuit app	Relational Frame Theory	Progressive Unlocking Sequential unlocking of levels based on progress Final levels require 7 consecutive smoke-free days Relapse support encourages the repetition of preparatory tasks
Bricker et al.^22^ 2020 USA	SCAMPI program	Behavior Change Wheel Framework	Leaderboards and Accountability Ranking board for competition Focus on the longest continuous smoking abstinence
Pallejà-Millán et al.^23^ 2020 Spain	Tobbstop app	Not stated	Metaphorical Gameplay and Rewards Detoxify and improve a polluted island Visual progress with island enhancements Rewards to motivate sustained efforts
Peek et al.^24^ 2021 Australia	My QuitBuddy app	Not stated	Milestone Recognition and Progress Tracking Trophies for continuous abstinence Progress tracker with financial savings and health benefits
Houston et al.^25^ 2022 USA	Take A Break (TAB)	Social Cognitive Theory	Leaderboards and Rewards Reward points with leaderboard rankings Tiered medals and gift cards for top performers
Schnall et al.^26^ 2022 USA	Lumme Quit Smoking app	Cognitive Behavioral Therapy	Milestone Recognition and Progress Tracking Badges or trophies for abstinence milestones Visual tracking of financial savings and health improvements
Marler et al.^27^ 2022 USA	Pivot	Cognitive Behavioral Therapy and Self-Determination Theory	Educational Game-Based Pathways Educational games structured into 4 tracts: Learn, Reduce, Prepare to Quit, and Maintain My Quit Designed to accommodate users at various readiness levels Participants can focus on self-awareness, plan creation, quit attempts, or maintenance, and navigate between tracts to access the most relevant content
Webb et al.^28^ 2022 UK	Quit Genius	Cognitive Behavioral Therapy	Milestone Recognition and Progress Tracking Achievements and rewards for reaching milestones Visual feedback on progress, including health improvements and money saved Streaks for daily goals and interactive quizzes to maintain engagement
Chen et al.^29^ 2024 China	CBT-based app	Cognitive Behavioral Therapy	Storytelling and Progress Tracking Gamified quitting journey with a dynamic storyline Achievement tracking and recognition Visual tracking of health and financial progress

In addition to gamification elements, many interventions were informed by established behavioral theories, which guided their design and implementation. Eleven studies explicitly referenced a theoretical framework, with Cognitive Behavioral Therapy (CBT) being the most frequently applied approach^15,21,26,27,29,30^. Several studies were based on Social Cognitive Theory, emphasizing the role of behavioral modelling and reinforcement^17,25^. Other theories used included Acceptance and Commitment Therapy^20^, Transactional Analysis Theory^19^, Relational Frame Theory^22^, and the Behavior Change Wheel Framework^21^. One study incorporated both Cognitive Behavioral Therapy and Self-Determination Theory, integrating psychological autonomy and motivation-based strategies into its design^27^.

### Meta-analysis of the effectiveness of gamification-based smoking cessation interventions

**Figure 2:**
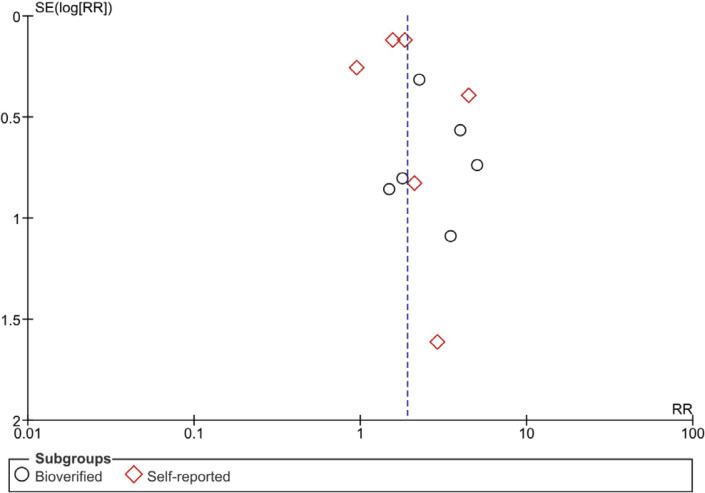
Funnel plot for visual assessment of publication bias in included studies

A meta-analysis was conducted to evaluate the effectiveness of gamification-based smoking cessation interventions. To minimize heterogeneity, we compared intervention effectiveness based on follow-up duration of the individual studies into short-term (<6 months), and long-term (≥6 months)^12^. The effects on tobacco cessation across the included studies are generally consistent and favor intervention over the non-intervention group, showing a statistically significant difference. Heterogeneity across studies was moderate, with I^2^ values ranging from 22% to 75%. A random-effects model was used due to variability in intervention designs and study populations. Visual evaluation of publication bias revealed asymmetrical distribution of the funnel plots, as shown in Figure 2.

### Smoking abstinence before six-month follow-up

**Figure 3:**
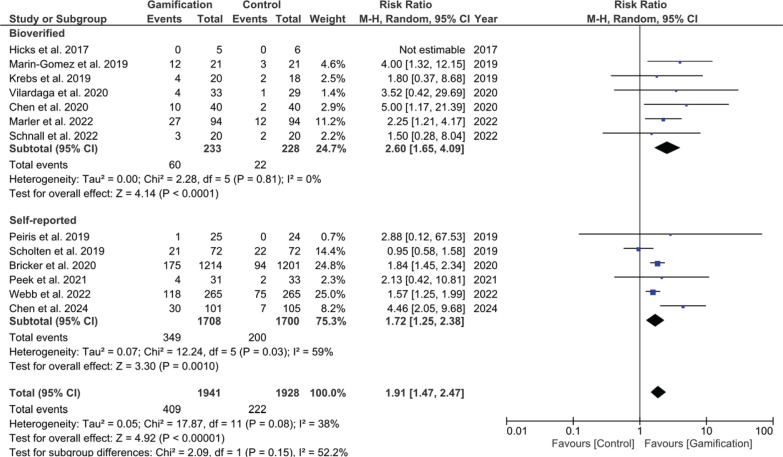
Forest plot of meta-analysis for short-term smoking cessation <6 months with sub-group analysis based on mode of verification

The pooled results from studies assessing smoking abstinence before six months showed a significant effect of gamification-based interventions. The overall relative risk (RR) was 1.91 (95% CI: 1.47–2.47, p<0.001), favoring gamified interventions. Subgroup analysis was performed based on the method of smoking abstinence verification (biochemically verified vs self-reported) and both significantly favored the intervention group, as shown in Figure 3. A formal test for subgroup differences showed no statistically significant interaction between groups (χ^2^=2.09, df=1, p=0.15, I^2^=52.2%), indicating that the effectiveness of gamification-based interventions did not differ significantly according to the method used to assess smoking abstinence. Sensitivity analysis, excluding high-risk-of-bias studies, confirmed the robustness of the findings (RR=1.70; 95% CI: 1.22–2.37, p=0.003).

### Smoking abstinence at or after six-month follow-up

**Figure 4:**
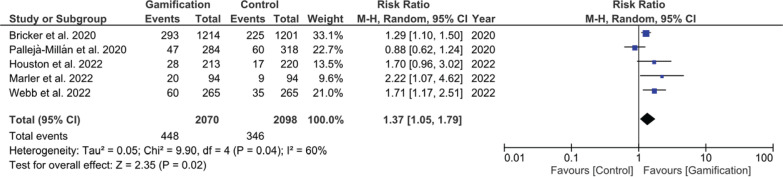
Forest plot of meta-analysis for long-term smoking abstinence (≥6 months)

Long-term smoking abstinence outcomes, assessed at six months or later, demonstrated sustained effectiveness of gamification-based interventions. The pooled RR was 1.37 (95% CI: 1.05–1.79, p=0.02), as shown in Figure 4, supporting the long-term impact. We did not proceed with subgroup analysis following mode of verification as only one study had self-reported smoking abstinence, while the remaining studies used bio-verification.

### Overall continuous abstinence since quit date

**Figure 5:**
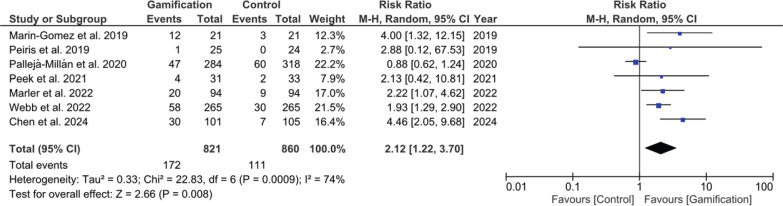
Forest plot of meta-analysis for overall continuous abstinence since quit date

Seven studies reported continuous abstinence from quit date. The pooled RR was 2.12 (95% CI: 1.22–3.770, p=0.008), shown in Figure 5, supporting the impact of gamified interventions on continuous abstinence.

## DISCUSSION

This systematic review and meta-analysis synthesized evidence from 15 randomized controlled trials (RCTs) evaluating the effectiveness of gamification in smoking cessation interventions, focusing primarily on smoking abstinence outcomes. The pooled findings indicate that interventions incorporating gamification elements resulted in significantly higher abstinence rates compared to those without gamification, irrespective of the mode of verification or time of assessment. Most interventions utilized mHealth platforms, particularly smartphone applications, which incorporated gamification elements such as leaderboards, progress tracking, milestone recognition, and reward systems. These features were shown to significantly enhance smoking abstinence rates compared to standard or non-gamified interventions.

The integration of gamification elements has previously been demonstrated to be effective in health behavioral interventions beyond smoking cessation, such as promoting physical activity among various populations^31^. Similarly, incorporation of gamification elements in interventions focusing on nutrition have successfully improved participants’ nutritional knowledge and encouraged healthier dietary behaviors^32^. These findings are consistent with the results of our review, reinforcing the incorporation of gamified interventions, especially those grounded in established behavioral theories, to significantly improve smoking cessation outcomes.

### Gamification elements and their impact on smoking abstinence

The success of gamified interventions in smoking cessation largely depends on the strategic integration of specific gamification elements designed to enhance user engagement, sustain motivation, and promote long-term adherence. These elements aim to address critical challenges such as high attrition rates and low participation^9^. However, in this study, assessing the effectiveness of individual gamification components on smoking cessation and abstinence was not feasible, as most applications employed multiple elements simultaneously and often in a randomized manner. Consequently, rather than isolating the impact of specific elements, we focused on identifying which gamification features were most used across interventions. The following discussion explores these frequently employed elements and their potential contributions to smoking cessation success.

*Rewards*

Rewards have consistently emerged as one of the most applied gamification elements in significant studies, providing extrinsic motivation that complements intrinsic goals. This aligns with Operant Conditioning, a behavioral theory emphasizing the role of positive reinforcement in promoting repeated engagement with desired behaviors^33^. By offering incentives in the form of points or achievement-based tokens, these interventions help bridge the gap between the immediate effort required to quit smoking and the long-term health benefits. This mechanism is particularly effective for individuals with lower intrinsic motivation. However, some form of rewards such as monetary incentives, vouchers, or gifts contingent on specific behaviors, may be perceived as too controlling and decrease intrinsic motivation. For example, a reward structure that requires users to meet rigid daily targets to progress or avoid penalties may shift the user’s focus from internal satisfaction to external validation. These differences in type of rewards may explain the conflicting outcomes in gamified interventions included in this review^34^.

*Competition through leaderboard display*

Leaderboards in smoking cessation interventions foster a sense of competition and accountability, making them particularly effective in creating social motivation^35^. By displaying participants’ progress and achievements, these leaderboards create a competitive environment that can significantly boost motivation and commitment to quitting smoking. The visibility of one’s standing among peers can trigger a range of psychological responses, from a desire to improve one’s position to a sense of pride in maintaining a high rank. This approach aligns with Social Cognitive Theory (SCT), which highlights the influence of peer dynamics and social comparison in enhancing motivation and shaping behavior^36^. By allowing participants to track their progress relative to others, leaderboards reinforce positive behaviors through external validation and accountability. This element not only promotes healthy competition but also sustains user engagement in smoking cessation efforts over time.

*Milestone recognition*

Progress tracking and milestone recognition serves as a powerful motivational tool in smoking cessation efforts by providing continuous feedback, highlights incremental achievements, and reinforces behavior change to smokers. Progress tracking aligns with Goal-Setting Theory, which underscores the importance of clear, measurable objectives in sustaining motivation^37^. By setting specific, achievable goals and regularly tracking progress towards these objectives, users can experience a sense of accomplishment and control over their smoking habits. Additionally, by visually presenting users with tangible evidence of their progress – such as days smoke-free, money saved, or improved health metrics – this element reduces the psychological distance to long-term goals and enhances self-efficacy, driving sustained engagement in smoking cessation efforts.

### Effectiveness across outcome measurement duration

The meta-analysis found that the effectiveness of gamified interventions varied based on the duration over which outcomes were assessed. In studies that examined smoking abstinence over a short-term period of six months, generally reported more robust intervention effects, whereas those with longer follow-up periods beyond six months demonstrated more attenuated results. For example, the study by Chen et al.^38^ found substantial improvements in smoking cessation rates during short-term follow-ups (RR=5.0, p=0.003), while the long-term investigation by Webb et al.^28^ demonstrated less pronounced, yet still statistically significant, relative risks (RR=1.71 at 52 weeks, p=0.005). These findings are consistent with previous research indicating that gamification’s initial attractiveness tends to decline as the novelty of the approach wears off^39^. However, interventions that incorporate elements intended to preserve user engagement, such as progress tracking systems and adaptive feedback, may offset the diminishing impact observed over time. The review highlighted that the incorporation of gamification elements such as progress monitoring, personalized reward, and social competition were identified as essential factors in facilitating smoking abstinence.

### Subgroup analysis: Biochemical verification versus self-reported abstinence

Further comparative subgroup analysis was performed to assess the efficacy of interventions that employed biochemical verification of smoking cessation against those that utilized self-reported abstinence. We found that while biochemical verification is considered the gold standard for assessing smoking abstinence^40^, the analysis revealed no statistically significant differences in the efficacy of interventions that utilized biochemical validation versus those relying on self-reported data. Although previous studies showed high proportion of self-reported quitters failing to confirm their abstinence biochemically^41-43^, our findings suggested that bio-verified outcome trials do not necessarily surpass self-reported measurement. However, given the high risk of social-desirability bias in self-reported questionnaires^44^, the SRNT Subcommittee on Biochemical Verification recommended to include biochemical verification for smoking abstinence in the study protocol whenever feasible^40^. Future research should focus on integrating self-reports with biochemical verification to improve the validity of findings.

### Sensitivity analysis

As the studies with high-risk bias were excluded in the sensitivity analysis, the effectiveness of gamified interventions was found to be more robust. While the exclusion of these studies resulted in a slight reduction in effect sizes, it did not significantly change the overall conclusions. The persistent presence of positive findings following sensitivity analysis indicates that gamified smoking cessation programs offer significant support, especially when it is integrated with effective behavioral strategies. These findings align with existing research that endorses digital health interventions for smoking cessation^4^.

### Strengths and limitations

This comprehensive review and meta-analysis synthesize the current evidence on gamified interventions for smoking cessation, encompassing diverse populations, gamification elements, and outcome measures. By incorporating both self-reported and biochemically verified abstinence outcomes, this analysis provides a thorough evaluation of effectiveness, underscoring the importance of objective outcome measures. The use of sensitivity analyses further strengthens the reliability of findings by accounting for potential biases in studies with a higher risk.

However, several limitations must be acknowledged. Variability in the definition of a ‘smoker’ across studies, along with significant heterogeneity in follow-up periods used to assess smoking cessation and abstinence, limits comparability. The reliance on self-reported measures in some studies introduces potential bias, while the inconsistent use of biochemical verification constrains the generalizability of results. Additionally, although gamification has shown promise in smoking cessation, the effectiveness of individual gamification elements could not be assessed, as most interventions employed multiple elements simultaneously, often in an overlapping or randomized manner. This lack of distinction made it challenging to determine which specific components contributed most to success. Future research should aim to address these limitations by standardizing outcome measures, improving study designs, and systematically evaluating the impact of individual gamification elements to optimize intervention effectiveness.

## CONCLUSIONS

This systematic review and meta-analysis provide robust evidence that gamification-based interventions significantly enhance smoking cessation outcomes. By synthesizing findings from 15 randomized controlled trials, we demonstrate that integrating gamification elements leads to markedly higher smoking abstinence rates compared to non-gamified interventions. However, our findings also reveal that these effects are more pronounced in the short-term, particularly within the first six months, suggesting that long-term engagement remains a critical challenge. This decline in effectiveness over time may be attributed to a novelty effect that diminishes user participation as engagement wanes. To ensure lasting behavioral change, future interventions must go beyond initial engagement strategies and focus on sustaining motivation and adherence over time.

The potential of gamification in smoking cessation extends beyond immediate outcomes. By identifying and optimizing the most effective gamification elements, future research can refine intervention designs to maximize long-term impact. As digital health innovations continue to evolve, integrating evidence-based gamification strategies presents a unique opportunity to revolutionize smoking cessation efforts, making them more engaging, scalable, and effective. Moving forward, the power of gamification can be harnessed not just as a tool for short-term success, but as a catalyst for sustained smoking cessation and broader tobacco control efforts.

## Supplementary Material



## Data Availability

The data supporting this research can be found in the Supplementary file.

## References

[1] World Health Organization. WHO report on the global tobacco epidemic 2021: addressing new and emerging products. World Health Organization; 2021. Accessed April 10, 2025. https://iris.who.int/bitstream/handle/10665/343287/9789240032095-eng.pdf?sequence=1

[2] Reitsma MB, Flor LS, Mullany EC, Gupta V, Hay SI, Gakidou E. Spatial, temporal, and demographic patterns in prevalence of smoking tobacco use and initiation among young people in 204 countries and territories, 1990-2019. Lancet Public Health. 2021;6(7):e472-e481. doi:10.1016/S2468-2667(21)00102-X 34051921 PMC8251503

[3] Cobos-Campos R, de Lafuente AS, Apiñaniz A, Parraza N, Llanos IP, Orive G. Effectiveness of mobile applications to quit smoking: Systematic review and meta-analysis. Tob Prev Cessat. 2020;6:62. doi:10.18332/tpc/127770 33241162 PMC7682489

[4] Whittaker R, McRobbie H, Bullen C, Rodgers A, Gu Y, Dobson R. Mobile phone text messaging and app-based interventions for smoking cessation. Cochrane Database Syst Rev. 2019;10(10):CD006611. doi:10.1002/14651858.CD006611.pub5 31638271 PMC6804292

[5] Zhang M, Wolters M, O’Connor S, Wang Y, Doi L. Smokers’ user experience of smoking cessation apps: A systematic review. Int J Med Inform. 2023;175:105069. doi:10.1016/j.ijmedinf.2023.105069 37084673

[6] Guo N, Luk TT, Wu YS, et al. Effect of mobile interventions with nicotine replacement therapy sampling on long-term smoking cessation in community smokers: A pragmatic randomized clinical trial. Tob Induc Dis. 2023;21:44. doi:10.18332/tid/160168 36969982 PMC10037427

[7] Fang YE, Zhang Z, Wang R, et al. Effectiveness of ehealth smoking cessation interventions: Systematic review and meta-analysis. J Med Internet Res. 2023;25:e45111. doi:10.2196/45111 37505802 PMC10422176

[8] Rajani NB, Bustamante L, Weth D, Romo L, Mastellos N, Filippidis FT. Engagement with gamification elements in a smoking cessation app and short-term smoking abstinence: Quantitative assessment. JMIR Serious Games. 2023;11:e39975. doi:10.2196/39975 36724003 PMC9932870

[9] Rajani NB, Mastellos N, Filippidis FT. Impact of gamification on the self-efficacy and motivation to quit of smokers: Observational study of two gamified smoking cessation mobile apps. JMIR Serious Games. 2021;9(2):e27290. doi:10.2196/27290 33904824 PMC8114162

[10] White JS, Toussaert S, Raiff BR, et al. Evaluating the impact of a game (inner dragon) on user engagement within a leading smartphone app for smoking cessation: Randomized controlled trial. J Med Internet Res. 2024;26:e57839. doi:10.2196/57839 39475840 PMC11561441

[11] Page MJ, McKenzie JE, Bossuyt PM, et al. The PRISMA 2020 statement: an updated guideline for reporting systematic reviews. BMJ. 2021;372:n71. doi:10.1136/bmj.n71 33782057 PMC8005924

[12] Piper ME, Bullen C, Krishnan-Sarin S, et al. Defining and measuring abstinence in clinical trials of smoking cessation interventions: An updated review. Nicotine Tob Res. 2020;22(7):1098-1106. doi:10.1093/ntr/ntz110 31271211 PMC9633719

[13] Deterding S, Sicart M, Nacke L, O’Hara K, Dixon D. Gamification. using game-design elements in non-gaming contexts. In: Proceedings of the CHI ’11 Extended Abstracts on Human Factors in Computing Systems. Association for Computing Machinery; 2011:2425-2428. doi:10.1145/1979742.1979575

[14] Sterne JAC, Savović J, Page MJ, et al. RoB 2: a revised tool for assessing risk of bias in randomised trials. BMJ. 2019;366:l4898. doi:10.1136/bmj.l4898 31462531

[15] Hicks TA Bs, Thomas SP, Wilson SM, Calhoun PS, Kuhn ER, Beckham JC. A preliminary investigation of a relapse prevention mobile application to maintain smoking abstinence among individuals with posttraumatic stress disorder. J Dual Diagn. 2017;13(1):15-20. doi:10.1080/15504263.2016.1267828 27918881 PMC5360513

[16] Marin-Gomez FX, Garcia-Moreno Marchán R, Mayos-Fernandez A, et al. Exploring efficacy of a serious game (Tobbstop) for smoking cessation during pregnancy: Randomized controlled trial. JMIR Serious Games. 2019;7(1):e12835. doi:10.2196/12835 30916655 PMC6456830

[17] Krebs P, Burkhalter J, Fiske J, et al. The QuitIT coping skills game for promoting tobacco cessation among smokers diagnosed with cancer: pilot randomized controlled trial. JMIR Mhealth Uhealth. 2019;7(1):e10071. doi:10.2196/10071 30632971 PMC6329892

[18] Peiris D, Wright L, News M, et al. A Smartphone app to assist smoking cessation among aboriginal australians: findings from a pilot randomized controlled trial. JMIR Mhealth Uhealth. 2019;7(4):e12745. doi:10.2196/12745 30938691 PMC6538311

[19] Scholten H, Luijten M, Granic I. A randomized controlled trial to test the effectiveness of a peer-based social mobile game intervention to reduce smoking in youth. Dev Psychopathol. 2019;31(5):1923-1943. doi:10.1017/S0954579419001378 31607279

[20] Vilardaga R, Rizo J, Palenski PE, Mannelli P, Oliver JA, Mcclernon FJ. Pilot randomized controlled trial of a novel smoking cessation app designed for individuals with co-occurring tobacco use disorder and serious mental illness. Nicotine Tob Res. 2020;22(9):1533-1542. doi:10.1093/ntr/ntz202 31667501 PMC7443597

[21] Chen J, Ho E, Jiang Y, Whittaker R, Yang T, Bullen C. Mobile social network-based smoking cessation intervention for Chinese male smokers: pilot randomized controlled trial. JMIR Mhealth Uhealth. 2020;8(10):e17522. doi:10.2196/17522 33095184 PMC7647814

[22] Bricker JB, Watson NL, Mull KE, Sullivan BM, Heffner JL. Efficacy of smartphone applications for smoking cessation: a randomized clinical trial. JAMA Intern Med. 2020;180(11):1472-1480. doi:10.1001/jamainternmed.2020.4055 32955554 PMC7506605

[23] Pallejà-Millán M, Rey-Reñones C, Barrera Uriarte ML, et al. Evaluation of the tobbstop mobile app for smoking cessation: cluster randomized controlled clinical trial. JMIR Mhealth Uhealth. 2020;8(6):e15951. doi:10.2196/15951 32589153 PMC7381259

[24] Peek J, Hay K, Hughes P, et al. Feasibility and acceptability of a smoking cessation smartphone app (My QuitBuddy) in older persons: pilot randomized controlled trial. JMIR Form Res. 2021;5(4):e24976. doi:10.2196/24976 33851923 PMC8082378

[25] Houston TK, Chen J, Amante DJ, et al. Effect of technology-assisted brief abstinence game on long-term smoking cessation in individuals not yet ready to quit: a randomized clinical trial. JAMA Intern Med. 2022;182(3):303-312. doi:10.1001/jamainternmed.2021.7866 35072714 PMC8787683

[26] Schnall R, Liu J, Alvarez G, et al. A smoking cessation mobile app for persons living with hiv: preliminary efficacy and feasibility study. JMIR Form Res. 2022;6(8):e28626. doi:10.2196/28626 35980739 PMC9437787

[27] Marler JD, Fujii CA, Utley MT, Balbierz DJ, Galanko JA, Utley DS. Outcomes of a comprehensive mobile smoking cessation program with nicotine replacement therapy in adult smokers: pilot randomized controlled trial. JMIR Mhealth Uhealth. 2022;10(11):e41658. doi:10.2196/41658 36257323 PMC9732762

[28] Webb J, Peerbux S, Smittenaar P, et al. Preliminary outcomes of a digital therapeutic intervention for smoking cessation in adult smokers: randomized controlled trial. JMIR Ment Health. 2020;7(10):e22833. doi:10.2196/22833 33021488 PMC7576529

[29] Chen S, Tang J, Wu C, Zhang G, Zhang J, Liao Y. Preliminary efficacy of a cognitive behavioral therapy-based smartphone app for smoking cessation in china: randomized controlled pilot trial. JMIR Form Res. 2024;8:e48050. doi:10.2196/48050 38498030 PMC10985609

[30] Webb J, Peerbux S, Ang A, et al. Long-term effectiveness of a clinician-assisted digital cognitive behavioral therapy intervention for smoking cessation: secondary outcomes from a randomized controlled trial. Nicotine Tob Res. 2022;24(11):1763-1772. doi:10.1093/ntr/ntac113 35470860 PMC9597001

[31] Mazeas A, Duclos M, Pereira B, Chalabaev A. evaluating the effectiveness of gamification on physical activity: systematic review and meta-analysis of randomized controlled trials. J Med Internet Res. 2022;24(1):e26779. doi:10.2196/26779 34982715 PMC8767479

[32] Suleiman-Martos N, García-Lara RA, Martos-Cabrera MB, et al. Gamification for the improvement of diet, nutritional habits, and body composition in children and adolescents: a systematic review and meta-analysis. Nutrients. 2021;13(7):2478. doi:10.3390/nu13072478 34371989 PMC8308535

[33] Mcleod S. Operant conditioning: What it is, how it works, and examples. Simply Psychology. Updated March 17, 2025. Accessed April 10, 2025. https://www.simplypsychology.org/operant-conditioning.html

[34] Lewis ZH, Swartz MC, Lyons EJ. What’s the point?: a review of reward systems implemented in gamification interventions. Games Health J. 2016;5(2):93-99. doi:10.1089/g4h.2015.0078 26812253

[35] Bovermann K, Bastiaens T. Using gamification to foster intrinsic motivation and collaborative learning: a comparative testing. Presented at: EdMedia: World Conference on Educational Media and Technology; June 25, 2018. Accessed April 10, 2025. https://www.learntechlib.org/primary/p/184321/

[36] Schunk DH, DiΒenedetto MK. Motivation and social cognitive theory. Contemp Educ Psychol. 2020;60:101832. doi:10.1016/j.cedpsych.2019.101832

[37] Tondello GF, Premsukh H, Nacke LE. A theory of gamification principles through goal-setting theory. Presented at: Proceedings of the 51st Hawaii International Conference on System Sciences; 2018:1118-1127. doi:10.24251/HICSS.2018.140

[38] Chen J, Ho E, Jiang Y, Whittaker R, Yang T, Bullen C. A mobile social network-based smoking cessation intervention for Chinese male smokers: protocol for a pilot randomized controlled trial. JMIR Res Protoc. 2020;9(9):e18071. doi:10.2196/18071 32945261 PMC7532454

[39] Seaborn K, Fels DI. Gamification in theory and action: A survey. Int J Hum Comput Stud. 2015;74:14-31. doi:10.1016/j.ijhcs.2014.09.006

[40] Benowitz NL, Bernert JT, Foulds J, et al. Biochemical verification of tobacco use and abstinence: 2019 update. Nicotine Tob Res. 2020;22(7):1086-1097. doi:10.1093/ntr/ntz132 31570931 PMC7882145

[41] Patrick DL, Cheadle A, Thompson DC, Diehr P, Koepsell T, Kinne S. The validity of self-reported smoking: a review and meta-analysis. Am J Public Health. 1994;84(7):1086-1093. doi:10.2105/ajph.84.7.1086 8017530 PMC1614767

[42] Piper ME, Cook JW, Schlam TR, et al. A randomized controlled trial of an optimized smoking treatment delivered in primary care. Ann Behav Med. 2018;52(10):854-864. doi:10.1093/abm/kax059 30212849 PMC6135958

[43] Scheuermann TS, Richter KP, Rigotti NA, et al. Accuracy of self-reported smoking abstinence in clinical trials of hospital-initiated smoking interventions. Addiction. 2017;112(12):2227-2236. doi:10.1111/add.13913 28834608 PMC5673569

[44] van de Mortel TF. Faking it: social desirability response bias in self-report research. Aust J Adv Nurs. 2008;25(4):40-48. Accessed April 10, 2025. https://www.ajan.com.au/archive/Vol25/Vol_25-4_vandeMortel.pdf

